# A simplified, 2-question grading system for evaluating abstracts in orthopedic scientific meetings: a serial randomization study

**DOI:** 10.2340/17453674.2024.40504

**Published:** 2024-04-17

**Authors:** Walter VAN DER WEEGEN, Jeroen C VAN EGMOND, Ruth E GEUZE, Taco GOSENS, Barbara SNOEKER, Rudolf W POOLMAN

**Affiliations:** 1 Department of Orthopedics, Sint Anna Ziekenhuis, Eindhoven; 2 Department of Orthopedics, Bravis Ziekenhuis, Roosendaal; 3 Department of Orthopedics, Elisabeth-Tweesteden Ziekenhuis, Tilburg; 4 Department of Clinical Epidemiology, Biostatistics and Bioinformatics, Amsterdam Medical Center, University of Amsterdam; 5 Department of Orthopedics, Leiden University Medical Center, Leiden; 6 Department of Orthopedics, Joint Research, OLVG, Amsterdam, the Netherlands

## Abstract

**Background and purpose:**

Efficient abstract scoring for congress presentation is important. Given the emergence of new study methodologies, a scoring system that accommodates all study designs is warranted. We aimed to assess the equivalence of a simplified, 2-question abstract grading system with a more complex currently used system in assessing abstracts submitted for orthopedic scientific meetings in a serial randomized study.

**Methods:**

Dutch Orthopedic Association Scientific Committee (DOASC) members were randomized to grade abstracts using either the current grading system, which includes up to 7 scoring categories, or the new grading system, which consists of only 2 questions. Pearson correlation coefficient and mean abstract score with 95% confidence intervals (CI) were calculated.

**Results:**

Analysis included the scoring of 195 abstracts by 12–14 DOASC members. The average score for an abstract using the current system was 60 points (CI 58–62), compared with 63 points (CI 62–64) using the new system. By using the new system, abstracts were scored higher by 3.3 points (CI 1.7–5.0). Pearson correlation was poor with coefficient 0.38 (P < 0.001).

**Conclusion:**

The simplified abstract grading system exhibited a poor correlation with the current scoring system, while the new system offers a more inclusive evaluation of varying study designs and is preferred by almost all DOASC members.

Medical conferences globally host thousands of presentations annually. Maintaining high standards of scientific quality necessitates a reliable and efficient selection process for abstracts. Previous literature has explored methods for optimizing this selection process and the performance of different abstract rating systems [1-5].

A 2007 study conducted by the Scientific Committee of the Dutch Orthopedic Association (DOASC) demonstrated strong interobserver agreement utilizing the International Society of the Knee (ISK) quality-of-reporting system for abstracts [6]. This system favors randomized controlled trials (RCTs), giving top scores in the “Design” category for “prospective” and in the “Control group” category for “matched and randomized” [6]. However, despite their high standing in the evidence-based medicine hierarchy, RCTs do not represent the apex of study design, which is made up of systematic reviews.

Furthermore, emerging study methodologies have revealed limitations in the current scoring system’s capacity to accommodate all types of study designs featured in submitted abstracts. For instance, mixed-methods designs, scoping reviews, and systematic reviews may not fit comfortably within the current system, potentially excluding valuable research from conference dissemination. While it is plausible to augment existing grading systems, this approach may result in a more convoluted and time-intensive scoring process.

Recognizing these constraints for every scientific congress committee, and the evolving landscape of medical research, we wanted to devise and validate a simplified abstract scoring system capable of accommodating a broader range of study designs used in medical and orthopedic research.

The aim of the study was to examine the correlation and agreement between the proposed, simplified abstract grading system (“New system”), and the current grading system (“Old system”) in assessing submitted abstracts.

We hypothesize that the newly developed, simplified abstract grading system will demonstrate comparable inter-rater agreement compared with the current grading system while accommodating a broader array of study designs in medical and orthopedic research.

## Methods

**Figure 1:**
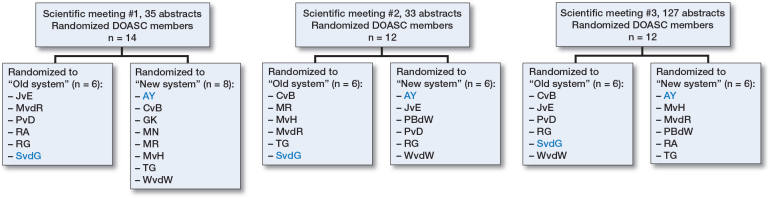
Flowchart of distribution of raters over the old and new systems after serial randomization. Blue are raters where cross-over did not occur.

We implemented a serial randomization study following the CONSORT statement to evaluate the scoring of abstracts submitted for 3 meetings held by the Dutch Orthopedic Society [7]. Systematic review abstracts were excluded from the analysis due to the current scoring system’s inability to evaluate this research type. All other abstracts were included. All DOASC members (n = 12–14) were eligible for study participation and were randomized 1:1 into 2 groups: 1 using the new scoring system, and the other utilizing the current system for abstract evaluation. This computer randomization process, managed by the DOASC coordinator (BS), was repeated before each meeting, after ensuring all abstracts were blinded. By repeating this randomization prior to each scientific symposium, most DOASC members were crossing over from one system to the other abstract grading system, albeit depending on chance whether this crossover occurred or not (Figure 1). This process of serial randomization allowed DOASC members to experience the usability of both grading systems and estimate the time taken to grade all submissions using each system. The reviewer composition evolved gradually, with a maximum turnover of 2 members per year. No formal training was provided for either scoring system; the current system was shared through an appendix, while the new system was introduced during a plenary meeting and first applied at the 2022 annual meeting.

### Abstract submission and selection process

2 Dutch Orthopedic meetings are held annually, both accepting abstract submissions until approximately 3 months prior to the meeting. Abstracts must not exceed 250 words and should include an introduction and study aim, methods, results, and conclusion. Abstract selection for oral presentation is determined by the quality of the abstract, the value of the results to the existing body of literature, and the number of submissions relative to the number of abstracts that can be selected for presentation.

The DOASC coordinator screened all submissions for format and language (Dutch only), subsequently anonymizing the abstracts before distribution to the DOASC members, along with a scoring system spreadsheet. All abstracts are independently graded by the 12–14 DOASC members, who then return their assessments to the DOASC coordinator for compilation into a ranked list. DOASC members with a conflict of interest do not score their own abstracts. As the DOASC group consists of a rather large group the possible influence on the results by individual DOASC members is mitigated.

### Current abstract scoring system

The current scoring system categorizes clinical studies, the most common type of abstract submissions, into 7 scoring categories, and basic science into 5 [6]. This system, providing a minimum score of 0 and a maximum score of 100, has been elaborated in a previous publication [6]. More details can be found in Table 1 (see Appendix).

### New simplified abstract scoring system

The new, simplified abstract scoring system was developed by DOASC members. A first draft was designed by WvdW and BS, which was then discussed with all DOASC members. It involves 2 questions: (1) “How would you assess the internal validity of the submitted abstract?” and (2) “Do the results contribute additional value to current knowledge?” A Numeric Rating Scale (NRS) was employed for response, ranging from 0 (very poor/no added value) to 10 (extremely good/very valuable) for each question. The scores from both questions were then combined to yield a total score, ranging from 0–20. To facilitate direct comparison, all total scores were multiplied by 5 to match the 100-point scale of the current scoring system.

### Statistics

Statistical analysis was performed using IBM SPSS Statistics for Windows (version 29.0, IBM Corp, Armonk, NY, USA). No sample size calculation was performed. We presumed that 3 rounds of abstracts would provide a sufficient number of abstracts. Descriptive statistics on abstract scores, including scatter plots, are presented. Floor and ceiling effects as well as clustering tendencies of both systems were evaluated with histogram plots of score frequencies. The differences in mean abstract scores between the two systems were compared and tested using a 2-sided independent Student t-test (α = 0.05) and with 95% confidence intervals (CI). A Pearson correlation coefficient was used to calculate the correlation between the current and new abstract scores. The inter-rater agreement, which refers to the consistency of ratings across DOASC members for each scoring system, was determined using intraclass correlation coefficients (ICC). The calculated ICC values range from –1 (indicating perfect disagreement) to +1 (indicating perfect agreement) [8]. An ICC between 0.75 and 0.90 was considered good and < 0.50 poor [9]. Following Koo and Li, we utilized a 2-way random effect to calculate the ICC in this study [9]. The overlap in the top 25% of scored abstracts from both scoring systems was compared for each of the 3 orthopedic meetings. No sub-analysis was performed. Finally, all DOASC members were asked which abstract scoring system was preferred.

### Ethics, registration, funding, and disclosures

The authors have no funding or financial support to declare. We were not able to register our study protocol, since our study is slightly different from a standard randomized trial. All authors have no conflict of interests to declare. Complete disclosure of interest forms according to ICMJE are available on the article page, doi: 10.2340/17453674.2024.40504

## Results

### Included abstracts and scoring analysis

**Figure 2:**
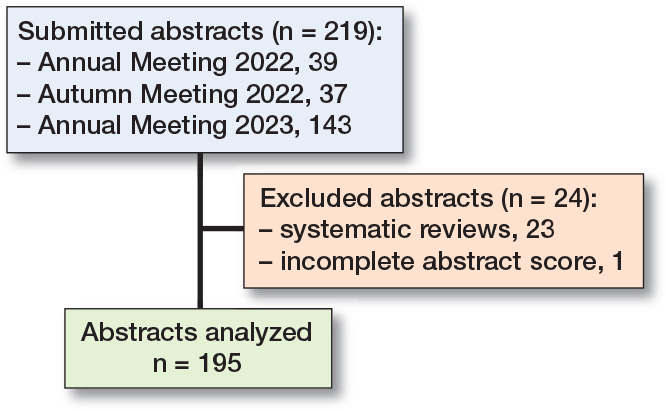
Flowchart of abstract inclusion.

Abstracts submitted to the 2022 Dutch Orthopedic Society Annual Meeting (n = 39), the 2022 Autumn Meeting (n = 37), and the 2023 Annual Meeting (n = 143) were included in our study (Figure 2).

Of the 219 submitted abstracts, we excluded 24 abstracts of which 23 were systematic reviews and 1 was incompletely scored, leaving 195 abstracts available for scoring analysis. All types of designs were represented and the category “other” includes studies such as validation studies, prediction models, consensus statement, and machine learning (Table 2). For the 2022 Autumn Meeting, 2 of the raters randomized to the current system did use the new system instead. At the 2023 Annual Meeting, 3 raters randomized to the current rating system failed to complete their abstract review process (Figure 1). The average score for an abstract using the current system was 60 points (SD 12.5, CI 58–62), compared with 63 points (SD 6.9, CI 62–64) using the new system. The mean difference in score per abstract was 3.3 points higher (SD 11.7, CI 1.7–5.0) when utilizing the new system, which was a statistically significant difference.

**Table 2:** Included study designs

Study design	n
Retrospective cohort study	69
Randomized controlled trial	20
Prospective cohort study	38
Cross-sectional cohort study	9
Questionnaire	11
Qualitative study	6
Basic science	20
Systematic review	23
Miscellaneous	23
Total	219

**Figure 3:**
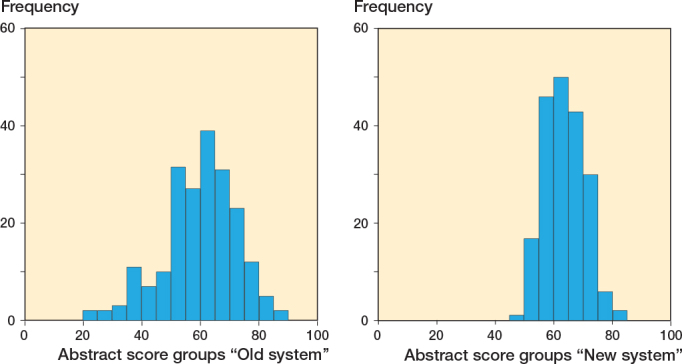
Histogram of observed scoring frequencies with the old and new systems.

**Figure 4:**
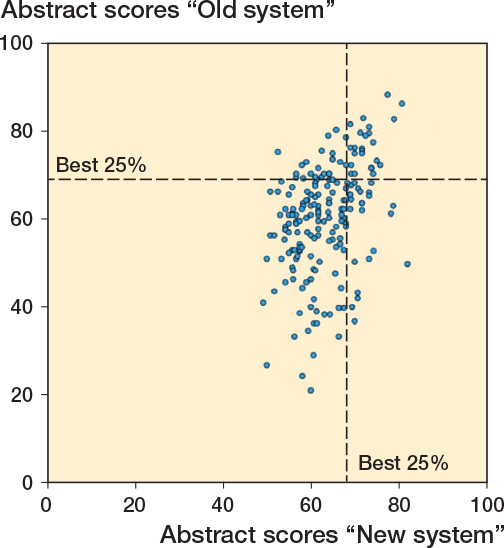
Scatter plot comparing the old with the new scoring system.

There were no strong floor or ceiling effects observed with either system. The major part of the scores was between 50 and 75 with a peak at 60 in both, but the range was larger in the current system, 20–90 versus 47.5–82.5 (Figure 3). A scatter plot of these results is presented in Figure 4. A poor, statistically significant correlation was observed between the scores from the current and new systems (Pearson correlation 0.38, P < 0.001).

### Interrater agreement, scoring variability, and abstract ranking

The intra-class correlation coefficient (ICC) for raters using the current system was 0.28 (CI 0.13–0.49), while the ICC for raters employing the new system was 0.09 (CI –0.008 to 0.27).

For each abstract, we calculated the standard deviation of scores assigned by both the current and new systems, with the mean of all these standard deviations being 10.1 for the current system and 11.1 for the new system, meaning that even if a system is consistent in its own ratings (indicated by a low standard deviation), there can still be little agreement between different raters (indicated by a low ICC).

At the 2022 Annual Meeting, the top 25% of abstracts (n = 9) as ranked by the current system overlapped with 6 of the top 25% ranked by the new system. At the 2022 Autumn Meeting, this overlap was 4 of the top 8 abstracts, and at the 2023 Annual Meeting, it was 18 of the top 32 abstracts. Overall, 28 of 49 were ranked as in the top 25% by both methods (Figure 4).

Among the 23 systematic reviews, which were excluded from score comparison as they could only be scored with the new system, 5 received sufficiently high scores using the new system to be included in the top 25% of abstracts.

### Preference for the new system

Among the 13 DOASC members, 12 expressed a preference for the new system, largely due to its usability across various study designs and its time efficiency. On average, the new system was estimated to be 60% quicker than the current system. Suggestions were made to include a manual on scoring internal validity. One DOASC member found the current system more systematic, suggesting that it offered superior guidance on abstract grading.

## Discussion

Our study focused on assessing equivalence between a newly developed, simplified grading system with he current, more intricate one used for evaluating abstracts for scientific medical conferences, a process that is crucial yet time demanding.

Our findings demonstrate a poor correlation between the 2 grading systems, with the simplified method receiving favorable feedback from the DOASC members due to its reduced time consumption and improved usability. Furthermore, both the current and new system did not show strong floor or ceiling effects. For enhancing agreement between different raters, the inclusion of a manual or guideline for grading the validity of submitted abstracts is suggested.

Notably, the ICCs for both the current and the new system in our study indicated poor inter-rater reliability, contrary to previous reports demonstrating good to excellent ICC scores for the current system (0.68–0.96) [6]. However, the standard deviation in scores for each abstract, ranging from 10.1 (current system) to 11.1 (new system) on a 100-point scale, suggests an acceptable dispersion of scores.

To evaluate the new scoring system, 23 of the 219 submitted abstracts had to be excluded from analysis as these were systematic reviews, which cannot be scored with the current system that is only designed to score studies with a trial design. Furthermore, various remaining studies were also difficult to score with the current system based on their deviant study design, including 6 qualitative studies and 22 with alternative study designs (i.e., controlled interrupted time-series design). Therefore 23 + 6 + 22 = 51 abstracts of a total of 219 were difficult to score with the current system.

Interestingly, 57% of the top-ranked 25% of abstracts (n = 49) using the current system overlapped with those ranked similarly by the new system, raising questions as to whether introducing the new system would result in a different selection of abstracts for podium presentations. We presume that this indicates that now the emerging new study designs would be correctly assessed and selected, where these were previously underrated with the current system.

The scientific literature presents a multitude of abstract scoring systems. For instance, Mitchell et al. compared an existing 3-criteria system with a new 4-criteria system and found an improvement in inter-rater agreement [10]. In the non-randomized study of Rahbek et al. a VAS scoring system was compared with the ISK system, with poorer intra- and interrater agreement for the VAS system [5]. Other studies employed systems with varying numbers of categories and scoring ranges [2,3,11]. Our proposed system, with fewer categories, reduces the grading time per abstract, but may benefit from training and guidelines to improve inter-rater consistency.

An interesting and surprising finding was the lower ICC of 0.28 for using the current system as seen in this present study, compared with an ICC of 0.68 for this system presented in a previous study [6]. We cannot fully explain why the ICCs were lower than in the publication of 2007. Further research into both the old and new abstract scoring system should be conducted through a large group of reviewers to gain a better understanding of the ICC.

We hypothesized that, due to the emerging submission of studies with novel study designs, the ICCs decreased because the ISK scoring cannot score these studies properly. This is why we are searching for a better way of scoring the abstracts.

### Limitations

Our experiment is subject to several limitations. Due to the study design, we were not able to register our study protocol, because our study is slightly different from a standard randomized trial. Both the current and new grading systems displayed poor inter-rater reliability, as indicated by the ICC, suggesting that a lack of formal training on the scoring systems may have influenced the raters’ scoring and overall outcomes. The experiment also did not account for potential biases related to raters’ personal preferences or familiarity with certain research areas or methodologies. Furthermore, we were unable to include abstracts presenting systematic reviews. Finally, even though the simplified grading system was faster, we did not consider possible trade-offs between speed and the in-depth evaluation provided by the more complex system.

### Strengths

The strengths of our experiment lie in its robust design and comprehensive evaluation. We used a serial randomized design, which allowed for a direct comparison between the current and the new grading systems, increasing the validity of our findings. DOASC members involved with one of the abstracts are not supposed to review their own study. Moreover, the inclusion of a large number of raters (n = 12–14) who had different levels of proficiency in grading abstracts added to the robustness of our study, enhancing the generalizability of the results. Additionally, we examined the preferences of the raters for each system, thereby capturing not only the quantitative differences in scoring but also the qualitative user experience. Finally, our study spanned 3 different meetings, increasing the diversity and volume of the abstracts included in the study, which further bolsters the reliability of our findings.

### Conclusion

The proposed simplified abstract rating system (new system) correlates poorly with the current (old system) in assessing submitted abstracts, and is preferred by the majority of DOASC members. The simplified abstract grading system accommodates a broader array of study designs.

## Notes

WvdW designed the study, performed analysis, and he wrote and revised the manuscript. JE designed the study, supported the analysis, and wrote and revised the manuscript. RG, TG, and BS designed the study and critically reviewed the manuscript. RP designed the study, supported the analysis, and critically reviewed the manuscript.

Handling co-editors: Jeppe Vejlgaard Rasmussen and Robin Christensen

*Acta* thanks Thomas Jakobsen nd Maziar Mohaddes for help with peer review of this manuscript.

## Appendix

**Table 2:** Old grading system

Scoring for abstracts of clinical studies (maximum 100 points)	
Baseline score	+50
1. Problem description	
clear	+5
vague	0
non-existent	−5
2. Design	
prospective	+5
retrospective	0
questionnaire only	−5
not specified	−10
3. Control group	
yes	0
multiple tests, one group	+5
matched	+5
randomized	+10
matched and randomized	+15
no control	−5
4. Material	
unique	+5
adequate (size, length of follow-up)	0
insufficient (or not described)	−5
5. Methods	
objective and valid	+10
described	0
not described	−5
6. Results	
unique	+10
new and important	+5
existing knowledge	0
not important or incoherent	−5
not presented	−10
7. Conclusions	
valid	0
not supported by results	−5
non-existent	−10

**Table:**

Scoring for abstracts of experimental studies ^a^ (maximum 100 points)	
Baseline score	+50
1. Problem description	
important problem	+5
clear	0
vague	−5
2. Animal experiments only:	
Design	
Control group	
yes	0
multiple, tests, same group	+5
matched	+5
randomized	+10
matched and randomized	+15
no control	−10
Methods	
objective and valid	+10
described	0
not described	−10
3. Anatomy and biomechanics only:	
Material (samples, specimens)	
unique	+10
adequate	0
insufficient	−10
Methods	
objective and valid	+15
well described (accuracy and technique	+5
described	0
not described	−10
4. Results	
unique	+10
new and important	+5
existing knowledge	0
not important	−5
incoherent with methods or wrong	−10
not presented	−15
5. Conclusions	
valid	+5
not supported by results	−5
non described	−10

a Laboratory experiments, anatomy, biomechanics, animal studies
